# Development of preoperative nomograms to predict the risk of overall and multifocal positive surgical margin after radical prostatectomy

**DOI:** 10.1186/s40644-024-00749-w

**Published:** 2024-08-08

**Authors:** Lili Xu, Qianyu Peng, Gumuyang Zhang, Daming Zhang, Jiahui Zhang, Xiaoxiao Zhang, Xin Bai, Li Chen, Erjia Guo, Yu Xiao, Zhengyu Jin, Hao Sun

**Affiliations:** 1 Department of Radiology, Zhejiang Cancer Hospital, Hangzhou Institute of Medicine (HIM), Chinese Academy of Sciences, No. 1 East Banshan Road, Gongshu District, Hangzhou, 310022 China; 2 Department of Radiology, Peking Union Medical College Hospital, Peking Union Medical College, Chinese Academy of Medical Sciences, No.1 Shuaifuyuan, Wangfujing Street, Dongcheng District, Beijing, 100730 China; 3 Department of Pathology, Peking Union Medical College Hospital, Peking Union Medical College, Chinese Academy of Medical Sciences, No.1 Shuaifuyuan, Wangfujing Street, Dongcheng District, Beijing, 100730 China; 4 National Center for Quality Control of Radiology, No.1 Shuaifuyuan, Wangfujing Street, Dongcheng District, Beijing, 100730 China

**Keywords:** Magnetic resonance imaging, Prostatic neoplasms, Risk assessment, Margins of excision, Nomogram

## Abstract

**Objective:**

To develop preoperative nomograms using risk factors based on clinicopathological and MRI for predicting the risk of positive surgical margin (PSM) after radical prostatectomy (RP).

**Patients and methods:**

This study retrospectively enrolled patients who underwent prostate MRI before RP at our center between January 2015 and November 2022. Preoperative clinicopathological factors and MRI-based features were recorded for analysis. The presence of PSM (overall PSM [oPSM]) at pathology and the multifocality of PSM (mPSM) were evaluated. LASSO regression was employed for variable selection. For the final model construction, logistic regression was applied combined with the bootstrap method for internal verification. The risk probability of individual patients was visualized using a nomogram.

**Results:**

In all, 259 patients were included in this study, and 76 (29.3%) patients had PSM, including 40 patients with mPSM. Final multivariate logistic regression revealed that the independent risk factors for oPSM were tumor diameter, frank extraprostatic extension, and annual surgery volume (all *p* < 0.05), and the nomogram for oPSM reached an area under the curve (AUC) of 0.717 in development and 0.716 in internal verification. The independent risk factors for mPSM included the percentage of positive cores, tumor diameter, apex depth, and annual surgery volume (all *p* < 0.05), and the AUC of the nomogram for mPSM was 0.790 in both development and internal verification. The calibration curve analysis showed that these nomograms were well-calibrated for both oPSM and mPSM.

**Conclusions:**

The proposed nomograms showed good performance and were feasible in predicting oPSM and mPSM, which might facilitate more individualized management of prostate cancer patients who are candidates for surgery.

## Introduction

Radical prostatectomy (RP) serves as the primary treatment for patients with localized prostate cancer (PCa) [1]. Positive surgical margin (PSM) in RP specimens could indicate an unfavorable prognosis, which has been demonstrated to increase the risk of biochemical recurrence and even cancer-specific mortality in PCa patients [2–4]. Multifocality, one of the characteristics of PSM, indicates a poorer prognosis with an even higher risk for biochemical recurrence [5, 6]. Therefore, preoperative prediction of the risk of PSM could help in optimal treatment decision-making and selection of the surgical procedure in patients with PCa [7, 8].

Many studies have investigated the correlation between preoperative clinicopathological factors and the status of surgical margin in patients who underwent RP, and found that factors such as clinical stage, Gleason score obtained from biopsy, and percentage of positive biopsy cores, are important risk factors for PSM [2, 9–11]. In addition, pelvic anatomy is also found to be associated with the risk of PSM [12–14]. For example, a narrower and deeper pelvis and higher prostate apex depth are more likely to result in PSM [12, 14]. The features based on MRI are another important category for PSM risk evaluation. Tumor location and size determined on MRI, Prostate Imaging-Reporting and Data System (PI-RADS) category of lesions, and extraprostatic extension (EPE) observed on MRI are the most widely investigated [15–17]. Although many studies have focused on finding independent risk factors for PSM, some studies have attempted to propose algorithms or nomograms to facilitate individualized risk prediction of PSM [18, 19]. However, these models have usually been based solely on clinicopathological data or certain imaging features, and a more comprehensive preoperative model including clinicopathological data and MRI features is needed. Additionally, while the available studies have focused on constructing nomograms for overall PSM risk prediction, the nomograms for certain characteristics of PSM have rarely been reported. To further tailor the management of PCa, developing nomograms for preoperative evaluation of multifocal PSM is also necessary in clinical practice.

Therefore, our study aimed to construct a preoperative nomogram with clinicopathological and MRI-based risk factors to predict the risk of PSM after RP in PCa patients, and a nomogram for the adverse characteristic of PSM—the multifocality—to facilitate the individual management of PCa.

## Materials and methods

### Patients

**Fig. 1:**
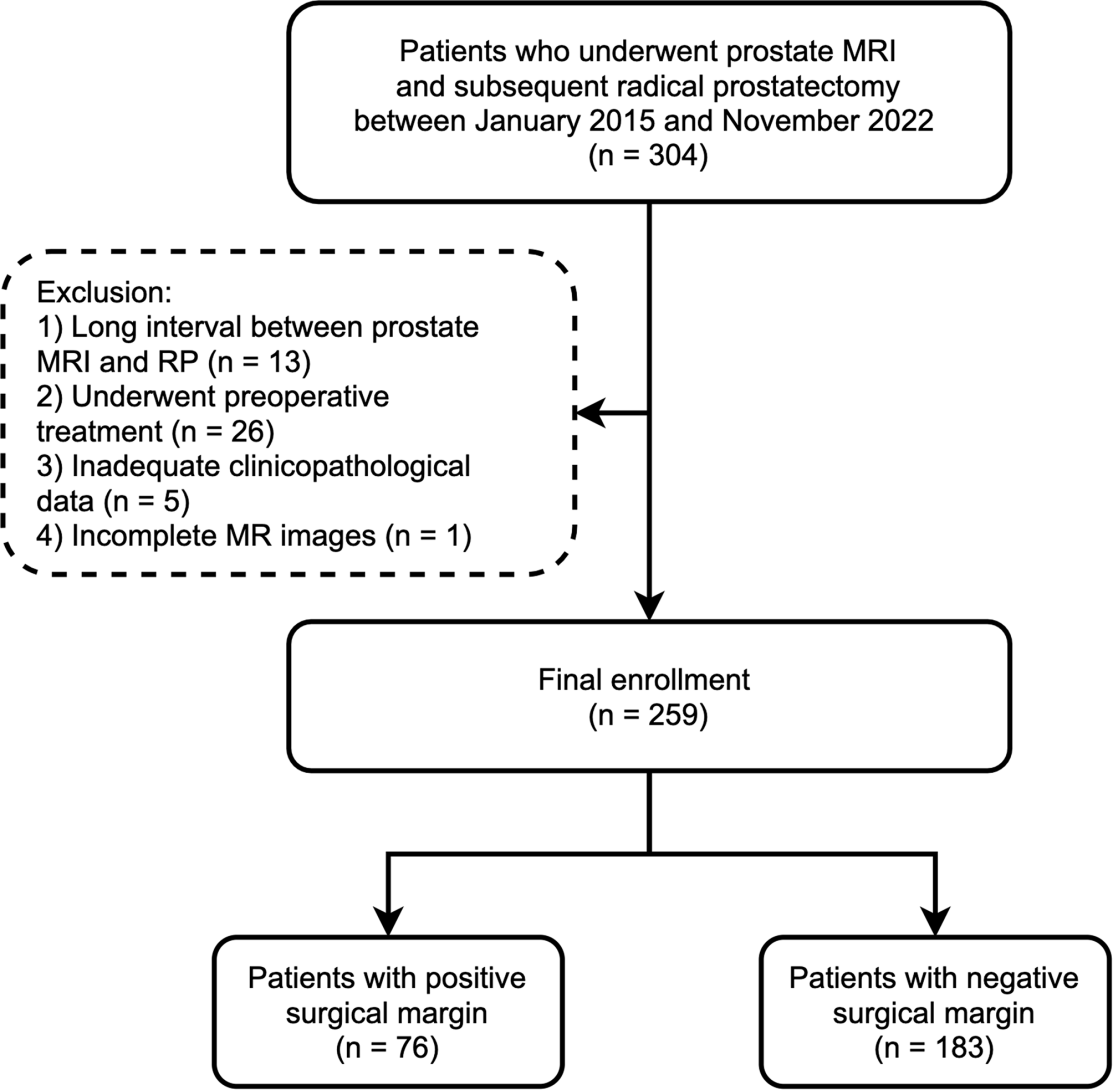
Flow diagram of patient selection in this study. RP = radical prostatectomy

This retrospective study was approved by the Institutional Research Ethics Committee, which waived the need for written informed consent. Consecutive patients who underwent prostate multiparametric MRI before laparoscopic RP at our facility between January 2015 and November 2022 were retrospectively enrolled. The following were the exclusion criteria: (1) more than six months between prostate MRI and RP; (2) patients who received preoperative treatments (radiation therapy, androgen-deprivation therapy, etc.); (3) patients without adequate clinicopathological data for analysis; and (4) sever artifacts or incomplete MRI exams. The study’s patient recruitment process is depicted in Fig. 1.

### Predictors

Potential predictors were selected based on a literature search and clinical experience. As the nomograms were aimed at evaluating the risk of PSM preoperatively, only the variables available before surgery were included in the analysis.

#### Clinicopathological predictors

Clinicopathological variables, including patient’s age, prostate-specific antigen (PSA) level, PSA density (PSAD), International Society of Urological Pathology (ISUP) grade from biopsy, clinical T stage, and surgery type (robot-assisted or not) were obtained from medical records. The percentage of positive biopsy cores was calculated by dividing the number of PCa cores by the total number of cores. The annual volume of surgery for surgeons was defined by the number of cases of RP for each surgeon.

#### MRI-based predictors

Two 3.0-T MRI scanners (GE750 [GE Healthcare] and Ingenia Elition [Philips]) were adapted to perform prostate MRI. The MRI protocol followed the PI-RADS recommendation and included T2-weighted images (T2WIs), diffusion-weighted images (DWIs), corresponding apparent diffusion coefficient (ADC) maps, and dynamic contrast-enhanced (DCE) images. The detailed parameters for obtaining MRI in this study are reported previously [20].

One radiologist (> 1000 prostate MRI images interpreted) who was blinded to the pathological results of RP reviewed all the MRI scans. The radiologist evaluated and included the following predictive factors: (1) features of prostate morphology and pelvic anatomy: prostate volume and prostate apex depth; (2) tumor-related risk factors: PI-RADS category of the index lesions, maximum tumor diameter measured on MRI, capsule contact length (CCL) of the lesions [21], frank EPE visible at MRI [22], and tumor’s distance to the proximal membranous urethra (UD). Frank EPE represents a clear breach of the prostate capsule due to the tumor extension into the periprostatic space. Prostate volume was calculated using the formula for a prostate ellipse.

### Reference standard

Following ISUP recommendations, the RP specimens were subjected to a routine pathological examination. The surface of the prostate gland was stained with ink, then fixed in formalin, and processed with a whole-mount thickness of 4 mm. The presence of PSM (overall PSM [oPSM]) at pathology and the multifocality of PSM (mPSM) were evaluated and recorded by experienced pathologists. The presence of cancer at the inked surgical margin was defined as PSM [23]. A single PSM was defined as only one positive location in the surgical specimen, while a multifocal PSM was considered when there was more than one positive location in the surgical specimen.

### Statistical analysis

First, univariate analysis with binary logistic regression was used to analyze the relationship between potential risk factors and oPSM and mPSM. The number of predictors in this study exceeded the binary logistic regression standard of events per variable ≥ 10; therefore, the LASSO method was used to screen candidate variables [24]. The LASSO method can handle multicollinearity; therefore, all the candidate variables were included in the LASSO regression model for further analysis. Based on the optimal lambda value, the predictive factors were selected for constructing a prediction model by the logistic regression method. Finally, the enhanced bootstrap method was used for internal validation [25]. The probability of risk for individual patients was calculated and visualized using a nomogram. To assess the predictive performance of the nomograms, the area under the receiver operating characteristic curve (AUC) was recorded, and the calibration curves of the models were further plotted using rms package in R to assess the model’s fitness to the observed results. *P* values < 0.05 were considered significant. All analyses were carried out using the R software (version 4.2.1; www.r-project.org). The code for analysis can be accessed through a public repository (https://github.com/LiliXu2022/R-for-nomogram.git).

## Results

### Patients’ demographic characteristics

The clinicopathological characteristics of the patient cohort are provided in Table 1. A total of 259 patients with a median age of 66 years (interquartile range [IQR], 62–70 years] were enrolled in the study. The median PSA level of 9.2 (6.4–14.8) ng/mL. PSM was found in 29.3% (76/259) of patients; 40 (52.6%) of them had multifocal PSM and 36 (47.4%) had solitary PSM.

**Table 1:** Demographic characteristics of patients

Variable	Population (*n* = 259)
Age (year)^*^	66 [62–70]
PSA (ng/mL)^*^	9.2 [6.4–14.8]
PSA density^*^	0.27 [0.17–0.45]
ISUP grade of biopsy (n [%])	
1	78 (30.1)
2	69 (26.6)
3	50 (19.3)
≥ 4	62 (23.9)
Percentage of positive biopsy cores (%)^*^	30.8 [15.4–50.0]
Prostate volume (mL)^*^	34.0 [26.0–47.0]
PI-RADS (n [%])	
2	2 (0.8)
3	26 (10.0)
4	117 (45.2)
5	114 (44.0)
Maximum tumor diameter on MRI (mm)^*^	14.1 [10.0–19.9]
Capsule contact length (mm)^*^	13.8 [7.4–22.5]
Frank EPE (n [%])	58 (22.4)
UD (mm)^*^	8.7 [4.8–13.1]
Apex depth (mm)	28.6 ± 6.0
Clinical T stage (n [%])	
cT1-2	201 (77.6)
cT3	58 (22.4)
Annual surgery volume (case)^*^	17 [7–45]
Robot-assisted surgery (n [%])	39 (15.1)
Positive surgical margin (n [%])	76 (29.3)
Solitary PSM	36 (47.4)
Multifocal PSM	40 (52.6)

Note—PSA = prostate-specific antigen, ISUP = International Society of Urological Pathology, PI-RADS = Prostate Imaging-Reporting and Data System, EPE = extraprostatic extension, UD = distance to the membranous urethra, PSM = positive surgical margin

^*^ Data are median (interquartile range [IQR]).

### Development and evaluation of the Nomogram for oPSM

**Fig. 2:**
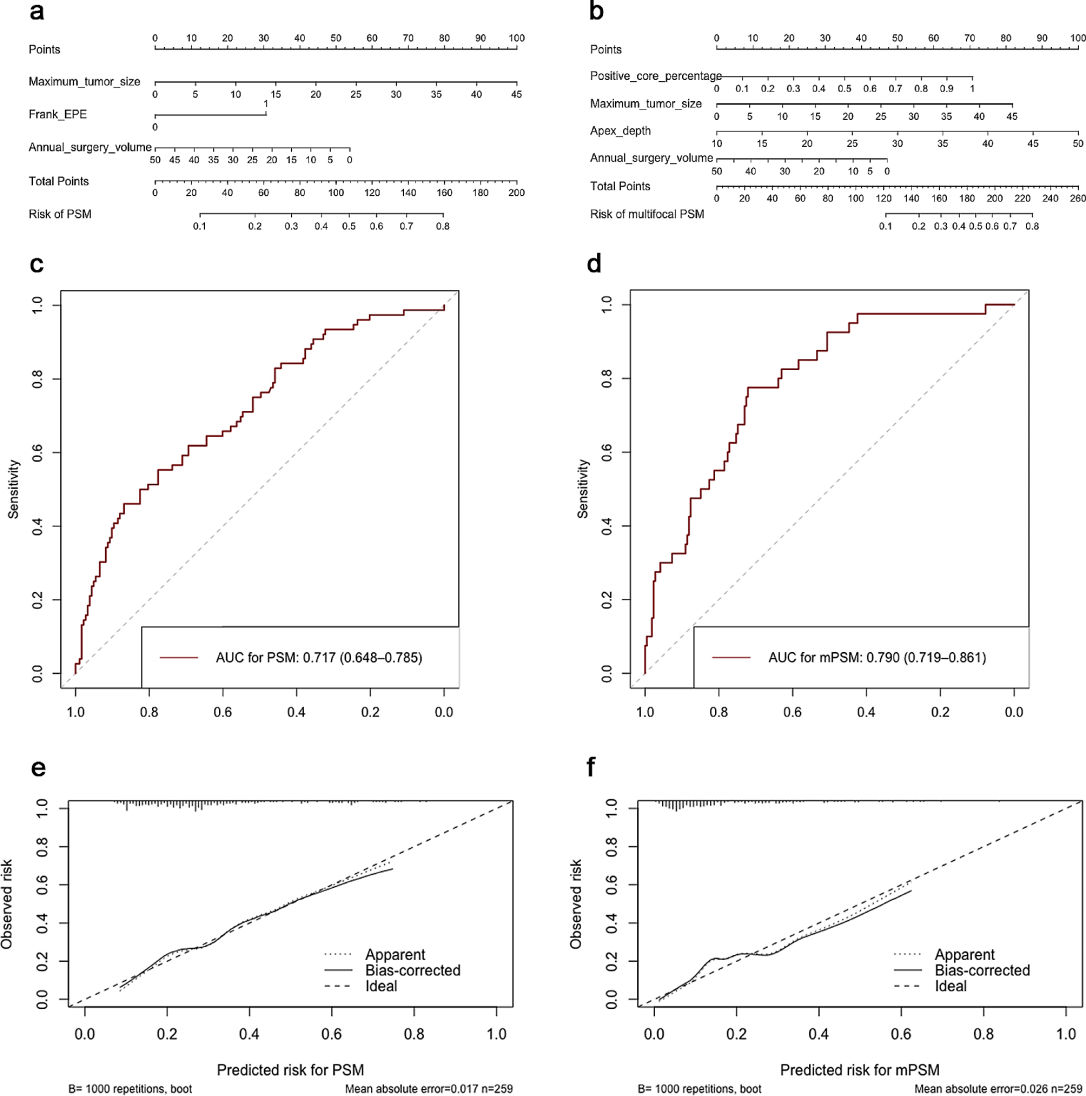
Nomograms, receiver operator characteristic curves, and calibration curves for predicting overall positive surgical margin (oPSM) and multifocal PSM (mPSM) after radical prostatectomy. The nomogram for oPSM included three variables, namely the maximum tumor diameter, frank EPE, and annual surgery volume (**a**). The area under the ROC curve (AUC) for the nomogram was 0.717 (95% confidence interval [CI]: 0.648–0.785) (**c**). The calibration curve analysis revealed a median absolute error of 0.017 after internal validation adjustment (**e**). The nomogram for mPSM contained four variables, including the positive core percentage of biopsy, maximum tumor diameter, apex depth, and annual surgery volume (**b**). The AUC for this nomogram was 0.790 (95% CI: 0.719–0.861) (**d**). The calibration curve analysis showed a median absolute error of 0.026 after internal validation (**f**)

In the univariate analysis, the ISUP grade of biopsy, percentage of positive cores, PI-RADS score, maximum tumor diameter, CCL, EPE, apex depth, cT, and annual surgery volume were significantly associated with oPSM (all *p* < 0.05). By the LASSO regression, three prediction factors were finally obtained and used to construct the prediction model by logistic regression. The variables included the maximum tumor diameter (odds ratio [OR], 1.061; 95% confidence interval [CI]: 1.012–1.112; *p* = 0.013), frank EPE (OR, 2.260; 95% CI: 1.020–5.008; *p* = 0.045), and annual surgery volume (OR, 0.972; 95% CI: 0.955–0.989; *p* = 0.001) (Table 2). The AUC of the model was 0.717 (95% CI: 0.648–0.785) (Fig. 2c), and after 1000-bootstrap internal verification, the AUC value was 0.716 (95% CI: 0.714–0.718). The nomogram was used to visualize the risk probability (Fig. 2a). Moreover, the calibration of the model was good with a mean absolute error of 0.017 and *p* = 0.972 by the Hosmer–Lemeshow test (Fig. 2e).

**Table 2:** Univariate and multivariate logistic regression analysis of predicting factors for positive surgical margin

Variables	Univariate			Multivariate		
	OR	95% CI	*p*	OR	95% CI	*p*
Age	1.033	0.988–1.081	0.163	-	-	-
PSA	1.017	0.996–1.039	0.104	-	-	-
PSA density	1.241	0.594–2.476	0.544	-	-	-
ISUP grade of biopsy						
1	reference	-	-	-	-	-
2	1.266	0.591–2.725	0.542	-	-	-
3	1.395	0.610–3.166	0.425	-	-	-
≥ 4	2.768	1.339–5.863	**0.007**	-	-	-
Percentage of positive cores	8.049	2.415–27.859	**0.001**	-	-	-
Prostate volume	0.994	0.980–1.008	0.428	-	-	-
PI-RADS						
≤ 3	reference	-	-	-	-	-
4	3.004	0.965–13.235	0.089	-	-	-
5	4.861	1.582–21.283	**0.014**	-	-	-
Maximum tumor diameter	1.080	1.042–1.121	**< 0.001**	1.061	1.012–1.112	**0.013**
Capsule contact length	1.044	1.020–1.069	**< 0.001**	-	-	-
Frank EPE	3.980	2.162–7.402	**< 0.001**	2.260	1.020–5.008	**0.045**
UD	0.975	0.931–1.016	0.245	-	-	-
Apex depth	1.050	1.004–1.101	**0.037**	-	-	-
Clinical T stage	2.023	1.092–3.719	**0.024**	-	-	-
Annual surgery volume	0.977	0.961–0.992	**0.003**	0.972	0.955–0.989	**0.001**
Robot-assisted surgery	1.629	0.788–3.286	0.178	-	-	**-**

Note—OR = odds ratio, CI = confidence interval, PSA = prostate-specific antigen, ISUP = International Society of Urological Pathology, PI-RADS = Prostate Imaging-Reporting and Data System, EPE = extraprostatic extension, UD = distance to the membranous urethra

Data in bold indicates *p* < 0.05

### Development and evaluation of the Nomogram for mPSM

In the univariate analysis, the percentage of positive cores, maximum tumor diameter, CCL, frank EPE, UD, apex depth, cT, and annual surgeon volume were noted to be significantly associated with mPSM (all *p* < 0.05). After LASSO regression for variable screening and logistic regression for model development, the final model included the percentage of positive cores (OR, 11.061; 95% CI: 2.078–58.880; *p* = 0.005), maximum tumor diameter (OR, 1.064; 95% CI: 1.014–1.116; *p* = 0.011), apex depth (OR, 1.089; 95% CI: 0.947–0.990; *p* = 0.005), and annual surgery volume (OR, 0.968; 95% CI: 0.947–0.990; *p* = 0.005) (Table 3). The AUC of the model was 0.790 (95% CI: 0.719–0.861), and after 1000-bootstrap internal verification, the AUC value was 0.790 (95% CI: 0.788–0.793) (Fig. 2d). The nomogram for mPSM is depicted in Fig. 2b. The model’s calibration curve showed a good calibration with a mean absolute error of 0.026 and *p* = 0.914 by the Hosmer–Lemeshow test (Fig. 2f).

**Table 3:** Univariate and multivariate logistic regression analysis of predicting factors for multifocal positive surgical margin

Variables	Univariate			Multivariate		
	OR	95% CI	*p*	OR	95% CI	*p*
Age	1.052	0.994–1.115	0.085	-	-	-
PSA	1.011	0.984–1.034	0.391	-	-	-
PSA density	1.268	0.494–2.767	0.577	-	-	-
ISUP grade of biopsy						
1	reference			-	-	-
2	0.892	0.321–2.404	0.821	-	-	-
3	0.927	0.297–2.681	0.891	-	-	-
4	2.365	0.999–5.836	0.054	-	-	-
Percentage of positive cores	23.035	5.279–107.866	**< 0.001**	11.061	2.078–58.880	**0.005**
Prostate volume	0.991	0.971–1.009	0.372	-	-	-
PI-RADS						
≤ 3				-	-	-
4	3.971	0.752–73.375	0.191	-	-	-
5	7.200	1.415–131.650	0.058	-	-	-
Maximum tumor diameter	1.078	1.035–1.125	**< 0.001**	1.064	1.014–1.116	**0.011**
Capsule contact length	1.038	1.009–1.068	**0.009**	-	-	-
Frank EPE	2.810	1.356–5.729	**0.005**	-	-	-
UD	0.909	0.842–0.971	**0.008**	-	-	-
Apex depth	1.098	1.036–1.168	**0.002**	1.089	1.021–1.161	**0.009**
Clinical T stage	2.142	1.013–4.395	**0.041**	-	-	-
Annual surgery volume	0.974	0.952–0.994	**0.013**	0.968	0.947–0.990	**0.005**
Robot-assisted surgery	1.240	0.471–2.906	0.639	-	-	**-**

Note—OR = odds ratio, CI = confidence interval, PSA = prostate-specific antigen, ISUP = International Society of Urological Pathology, PI-RADS = Prostate Imaging-Reporting and Data System, EPE = extraprostatic extension, UD = distance to the membranous urethra

Data in bold indicates *p* < 0.05

## Discussion

Preoperative prediction of the risk of PSM could affect the optimal treatment decision-making in PCa patients. This study found a difference in the independent risk factors for oPSM and mPSM. The maximum tumor diameter and frank EPE observed on MRI, and the surgeon’s annual surgery volume were the independent risk factors for oPSM. As for predicting mPSM, the percentage of positive cores in the biopsy, maximum tumor diameter and prostate apex depth measured on MRI, and surgeon’s annual surgery volume were the independent risk factors. Based on the selected risk factors, the preoperative prediction models for oPSM and mPSM were constructed, with AUC of 0.717 and 0.790, respectively. Both models were well calibrated by calibration curve analysis.

Preoperative prediction of PSM risk using clinicopathological and MRI features is possible. In some previous studies, the risk of PSM was assessed, and appropriate nomograms were compiled using preoperative characteristics. Hao et al. [18] built a model to predict the likelihood of PSM after robotic laparoscopic RP using a retrospective single-center cohort. Their model integrated the ISUP score, PI-RADS score, and PSA, with an adjusted C-statistic of 0.727. Tian et al. [19] proposed a preoperative nomogram for predicting PSM, in which the positive core percentage in the biopsy, clinical stage, free PSA/total PSA, and age were included. This model showed comparable performance, with a C-index of 0.722 in the development process and 0.700 in the bootstrap validations. In our study, we also found a correlation between the ISUP grade of biopsy, percentage of positive cores, PI-RADS score, and the risk of PSM in the univariate analysis. Apart from those well-studied clinicopathological characteristics, we additionally included some lesion-related and anatomy-related risk factors on MRI and surgeon experience in the analysis. The multivariate analysis showed that only the maximum tumor diameter and frank EPE on MRI, and annual surgery volume were the significant predictors and were used to construct the prediction model. Surgeon experience was a risk factor correlated with the risk of PSM. According to a systematic review [26] of the impact of surgeon volume on the outcomes after RP, higher surgeon volumes for RP are associated with lower rates of PSM. Steinsvik et al. [27] reported that the OR of PSM was 3.73 (95% CI: 2.25–6.17) for low-volume surgeons and 1.83 for intermediate-volume surgeons compared with high-volume surgeons. Frank EPE on MRI is highly predictive of pathological EPE [22], which has been demonstrated to be an independent risk factor for PSM [17, 28, 29]. A larger tumor size measured on MRI usually indicates a higher tumor burden. Surgical procedures in these patients should be more careful to achieve the complete resection of the lesions. Additionally, some studies included the variables that were only available by pathology analysis of RP specimens, such as pathological stage, Gleason score, and EPE, to evaluate the risk of PSM [2, 30, 31]. However, considering the availability of these variables, these factors or models seem to be of lower clinical utility in preoperative prediction.

Multifocality is a specific classification in PSM and correlates with a poorer prognosis; however, studies that construct prediction models for mPSM are limited. Gandi et al. found that the surgeon’s experience was significantly associated with multifocal/> 3 mm PSM [32]. Zhou et al.’s study [31] included preoperative clinical characteristics, inflammation scores, and postoperative pathological characteristics for analysis, and the results showed that lymphocyte-to-monocyte ratio (OR, 1.179), PSA (OR, 3.5), perineural invasion (OR, 3.446), and pathological Gleason sum (OR, 3.931) were the significant risk factors for multifocal PSM (all *p* < 0.05). In Qu et al.’s study, smoking or drinking history, tPSA levels, f/tPSA, percentage of positive needles, and Gleason score (all *p* < 0.05) were the risk factors for mPSM [33]. Although these studies have found verified risk factors for mPSM, they seldom tried to develop a prediction model for mPSM using these risk factors. In our study, the risk factors for mPSM were slightly different from the risk factors for oPSM. Apart from the maximum tumor diameter on MRI and surgeon’s annual surgery volume, which correlated with both mPSM and oPSM, the independent risk factors for mPSM additionally included the percentage of positive cores and prostate apex depth. A higher percentage of positive cores indicates a higher tumor burden and a more diffuse pattern of cancer lesions; therefore, it correlates with a higher risk of mPSM. Anthropometric measurements on MRI are associated with operative difficulties in RP [34]. Chen et al. [14] also concluded that limited surgical working space resulting from a narrower and deeper pelvis could affect patient outcomes, with an increased PSM rate. A deeper prostate apex is also an indicator of limited workspace for surgeons, which was demonstrated to be an independent risk factor for PSM by Matikainen et al. [12]. Nevertheless, in our study, the apex depth was an independent risk factor for mPSM but not a risk factor for oPSM. Therefore, mPSM is not only related to the tumor burden but is also closely related to surgical difficulty. Furthermore, we constructed a prediction model for mPSM with an AUC of 0.790 and displayed it in the nomogram, which could facilitate the clinical application of our model.

This study has some limitations. First, owing to the retrospective nature, there might be some selection bias for the patient cohort. Second, surgeon experience could vary from institution to institution; therefore, whether the nomograms are applicable to other institutions needs to be further verified. Nevertheless, our study emphasizes the surgery experience among the potential risk factors for PSM, which should be considered for further relevant studies. Finally, the correlation between the model-predicted risk and the observed survival in PCa patients remains unknown, and this needs to be further investigated.

## Conclusions

In conclusion, this study developed individual nomograms to predict the risks of oPSM and mPSM. Both nomograms showed good diagnostic performance for preoperative evaluation of the risk of PSM and might facilitate more individualized management of PCa patients who are candidates for surgery.

## Data Availability

The datasets used and analyzed during the current study are available from the corresponding author on reasonable request.

## Abbreviations

AUC: Area under the curve
CCL: Capsule contact length
CI: Confidence interval
EPE: Extraprostatic extension
IQR: Intequartile range
ISUP: International Society of Urological Pathology
mPSM: Multifocal positive surgical margin
oPSM: Overall positive surgical margin
OR: Odds ratio
PCa: Prostate cancer
PI-RADS: Prostate Imaging-Reporting and Data System
PSA: Prostate-specific antigen
PSAD: Prostate-specific antigen density
PSM: Positive surgical margin
RP: Radical prostatectomy
UD: Distance to the proximal membranous urethra
